# Ovarian cancer revealed by paraneoplastic cerebellar degeneration: a case report

**DOI:** 10.11604/pamj.2014.18.2.2808

**Published:** 2014-05-01

**Authors:** Fadwa Elomrani, Imane Ouziane, Saber Boutayeb, Youssef Bensouda, Hind Mrabti, Hassan Errihani

**Affiliations:** 1 Department of Medical Oncology, National Institute of Oncology, Rabat, Morocco

**Keywords:** Paraneoplastic cerebellar degeneration, Paraneoplastic syndromes, ovarian cancer

## Abstract

The prevalence of paraneoplastic cerebellar degeneration (PCD) associated with gynecological cancer is rare. Here, we reported the first case of ovarian cancer revealed by PCD in our institute. we describe a 80- year –old Moroccan female presented with subacute vestibular and cerebellar syndromes, she had an inguinal lymphadenopathy,with high levels of Anti-YO. Rapid progression and absence of known etiologies point towards a probable paraneoplastic origin of the syndrome in this patient. The exact incidence of PNS among those diagnosed with cancer remains uncertain, it is important to report this cases in the literature to help early diagnosis and appropriate treatment, which are able to stabilize the neurological symptoms.

## Introduction

Paraneoplastic syndromes (PNS) refer to symptoms or signs resulting from damage to organs or tissues that are remote from the site of a malignant neoplasm or its metastases. Paraneoplastic syndromes can affect most organs and tissues. Most or all paraneoplastic neurologic disorders are immune-mediated. Immunologic factors appear important in the pathogenesis of PNS because antineuronal autoantibodies and T-cell responses against nervous system antigens have been defined for many of these disorders. The cancers causing paraneoplastic neurologic disorders are often asymptomatic or occult but sometimes it can be revealed before the PNS [1]. We report in this article the diagnosis of ovarian cancer revealed by vestibular and cerebellar syndromes.

## Patient and observation

A 80 year- old Moroccan female, followed for arteriel hypertension treated by calcium antagonists, who consulted in our hospital complaining from vertigo, vomiting and nystagmus. The clinical examination found cerebellar syndrome and inguinal lymphadenopathy in the right measuring 25 mm. The brain MRI and puncture of cerebrospinal fluid were normal, Pet scan showed a right inguinal lymphadenopathy. Biopsy of the lymph node revealed metastasis of a poorly differentiated adenocarcinoma, with an immuno histochemical study CK7 +, CK20-, HR-, TTF1-. A dosage of Anti-YO was positive and Anti-HU antibody was negative, and CA125 was high. We retained the diagnosis of inguinal metastasis from gynecological cancer revealed by paraneoplastic neurologic syndromes, she received chemotherapy Paclitaxel-Carboplatin and symptomatic treatment based on corticosteroids and antiemetics, with a good clinical outcome after the first course, disappearance of cerebellar syndrome but after the second cure recurrence of the cerebellar syndrome and alteration of the general condition, she died in the month following.

## Discussion

Paraneoplastic neurologic syndromes are seen in less than 1% of all cancers [2, 3]. Since 2008 in our hospital we have received approximately 120 cases of ovarian cancer per year,it was the first case of ovarian cancer revealed by cerebellar syndromes. Some cases of ovarian cancer are also associated with paraneoplastic neurologic syndromes,such as necrotizing myelopathy, Subacute cerebellar degeneration and neuromyopathy [4].

Paraneoplastic cerebellar degeneration (PCD) is one of the most common paraneoplastic presentation of cancer is characterized by severe pancerebellar dysfunction. The postmortem study shows extensive loss of Purkinje neurons with relative preservation of other cerebellar neurons. Inflammatory infiltrates in the deep cerebellar nuclei and brainstem are also identified in some patients [5].

PCD has been reported in association with hodgkin's lymphoma, lung cancer and gynecologic tumors, breast or ovarian cancer. The diagnosis is difficult because in most cases the neurological symptoms precede the detection of the tumor [6].

Neurological deficits are sometimes preceded by prodromal symptoms, such as a nausea ,vomiting a viral-like illness, that might be attributed to a peripheral vestibular process, These symptoms are followed by dysarthria, , dysphagia. and ataxia, initially ataxia is asymmetric in 40% of the patients. After a few months symptoms stabilize,but leaving patient with a major deficit. Most of the patients have diplopia, horizontal nystagmus, and half of them have rotatory or downbeating nystagmus [5–8]. CT and MRI studies are normal or demonstrate cerebellar atrophy particularly in the latter stages of the Disease [5].

Many patients with paraneoplastic syndromes have antibodies in their serum (and cerebrospinal fluid). The identification of these antibodies facilitated the diagnosis. Anti-Yo antibodies are present in the majority of patients with PCD and cancer of the ovary, breast, or other gynecological malignancies. In two thirds of anti-Yo PCD patients the neurological symptoms develop before the diagnosis of the tumor.like our patient [5]

There is no standard of care for PCD,Clinical experience suggests that treatment of the tumour is needed for stabilisation or symptom improvement. The use of intravenous immunoglobulins, steroids or plasmapheresis and cyclophosphamide did not modify the neurological outcome of patients whose tumours were successfully treated, the best treatment is to induce a complete response of the tumor [9, 10]. Patients with anti-Hu or anti-Yo antibodies had a severe neurological deficits and less likely to improve than patients with anti-Tr antibodies. Survival from time of diagnosis is significantly worse in patients with anti-Yo (median 13 months) or anti-Hu (median 7 months) than in patients with anti-Tr (median >113 months) (11).

## Conclusion

Elderly women with PCD should be investigated for gynecological cancer and be treated as soon as possible for a better quality of life, because the evolution of disease is very fast and the prognosis is poor.
